# Alginate Heterografted Copolymer Thermo-Induced Hydrogel Reinforced by PAA-g-P(boc-L-Lysine): Effects on Hydrogel Thermoresponsiveness

**DOI:** 10.3390/polym16243555

**Published:** 2024-12-20

**Authors:** Aikaterini-Ariadni Moschidi, Constantinos Tsitsilianis

**Affiliations:** Department of Chemical Engineering, University of Patras, 26500 Patras, Greece; a.moschidi@rptu.de

**Keywords:** heterograft copolymer, hydrogel, alginate, thermoresponsiveness, injectability, mechanical reinforcement

## Abstract

In this article, we report on the alginate heterografted by Poly(N-isopropyl acrylamide-co-N-tert-butyl acrylamide) and Poly(N-isopropyl acrylamide) (ALG-g-P(NIPAM86-co-NtBAM14)-g-PNIPAM) copolymer thermoresponsive hydrogel, reinforced by substituting part of the 5 wt% aqueous formulation by small amounts of Poly(acrylic acid)-g-P(boc-L-Lysine) (PAA-g-P(b-LL)) graft copolymer (up to 1 wt%). The resulting complex hydrogels were explored by oscillatory and steady-state shear rheology. The thermoresponsive profile of the formulations were affected remarkably by increasing the PAA-g-P(b-LL) component of the polymer blend. Especially, the sol-gel behavior altered to soft gel–strong gel behavior due to the formation of a semi-interpenetrating network based on the hydrophobic self-organization of the PAA-g-P(b-LL). In addition, the critical characteristics, namely T_c,thermothickening_ (temperature above which the viscosity increases steeply) and ΔT (transition temperature window), shifted and broadened to lower temperatures, respectively, due to the influence of the hydrophobic side chains P(b-LL) on the LCST of the PNIPAM-based grafted chains of the alginate. The effect of ionic strength was also examined, showing that this is another important factor affecting the thermoresponsiveness of the hydrogel. Again, the thermoresponsive profile of the hydrogel was changed significantly by the presence of salt. All the formulations showed self-healing capability and tolerance injectability, suitable for potential bioapplications in living bodies.

## 1. Introduction

Injectable hydrogels constitute a particular class of soft matter based on three-dimensional (3D) networks, mainly formed by the crosslinking of water-soluble macromolecules through noncovalent, dynamic covalent, and in some cases covalent interactions. These interactions have been designed to provide reversible sol-gel properties, enabling the material to be injected into living bodies before undergoing gelation, thus avoiding invasive surgery. Self-healing is another property that makes hydrogels injectable, since they can temporarily fluidize under shear stress during injection and subsequently recover their original mechanical properties promptly postinjection. Thus, the injectable hydrogels have been recognized as very promising platforms for biomedical applications including tissue engineering, payload delivery, cancer treatment, wound dressings, 4D printing, etc., attracting immense attention for research and development [1,2,3,4,5].

In order to provide sol-gel properties to hydrogels, design strategies have suggested the use of copolymers, in which stimuli-responsive associative segments have been integrated. These kinds of gelators form a 3D reversible network, triggered from a stimulus, e.g., pH and/or temperature [6,7,8,9,10]. In this case, change in a stimulus affects the responsive segments (potential stickers) of the polymeric gelator which abruptly change their interactions with water (e.g., hydrophilic to hydrophobic transition), inducing association, consequently forming the physical crosslinks of the resulting network. Given that the human body is thermoregulated at about 37 °C, temperature is one of the most advantageous triggers to design the so-called thermoresponsive associative gelators targeting biomedical applications [11,12]. For this purpose, polymeric segments exhibiting LCST phase behavior are used as potential stickers [13,14]. The network is formed upon increasing the temperature passing the LCST of the polymer that is transformed to hydrophobic, becoming associative. The key factor in this design is to regulate the LCST to be lower than the physiological temperature (37 °C), thus providing sol-gel properties, which leads to in situ gelling [15,16,17]. A representative polymer exhibiting LCST below 37 °C, suitable for biomedical applications, is poly(N-isopropyl acrylamide) (PNIPAM) and its derivatives [13,18,19].

On the other hand, the nature of the backbone polymeric chains, forming the elastic chains of the network, is important. Biomedical applications require biomacromolecules exhibiting biocompatibility, biodegradability, and other important properties that could mimic the extracellular matrix (ECM). The family of polysaccharides seems to fulfil these requirements [20,21,22]. These natural materials are the most prominent and extensively studied biomacromolecules and are susceptible to effortless chemical modification due to the functional groups (e.g., carboxy, hydroxy, amine) which they bear. A commonly used modification strategy is the grafting-to reaction with end-functional short polymeric chains, transforming them into associative graft copolymers. By choosing LCST-based grafting chains, thermoresponsive polysaccharides can be designed to create injectable hydrogels [23,24]. Alginate, an abundant natural linear polysaccharide, constituted of l-guluronate (G) and d-mannuronate (M) residues, has found numerous applications in biomedical science and engineering mainly due to easy gelation through ionic interactions with divalent cations [25]. Alginate-based graft copolymers, bearing PNIPAM-based side chains with tunable thermoresponsive properties, have been developed recently [26,27,28,29,30,31,32,33,34,35,36,37].

As has been shown, the hydrogel properties and performance, namely injectability, self-healing, and mechanical suitability at the conditions (pH, temperature, ionic strength) of the targeting site environment (e.g., physiological, tumor, wound), depend mainly on the sol-gel transition temperature (*T_gel_*), which can be tuned by various design factors. For instance, by incorporating hydrophobic monomers into the PNIPAM side chains of alginate-based graft copolymers, *T_gel_* decreases linearly with the content of the N-tert-butyl acrylamide (NtBAM) hydrophobic monomer, which allows fine-tuning of the sol-gel transition. Moreover, the shift of the transition to lower temperatures significantly affects the hydrogel elasticity and dynamics at the physiological temperature [32]. Another strategy relies on the selection of the nature of the LCST grafted side chains, which again controls the sol-gel transition and the elastic properties of the injectable hydrogel [37].

**Scheme 1:**
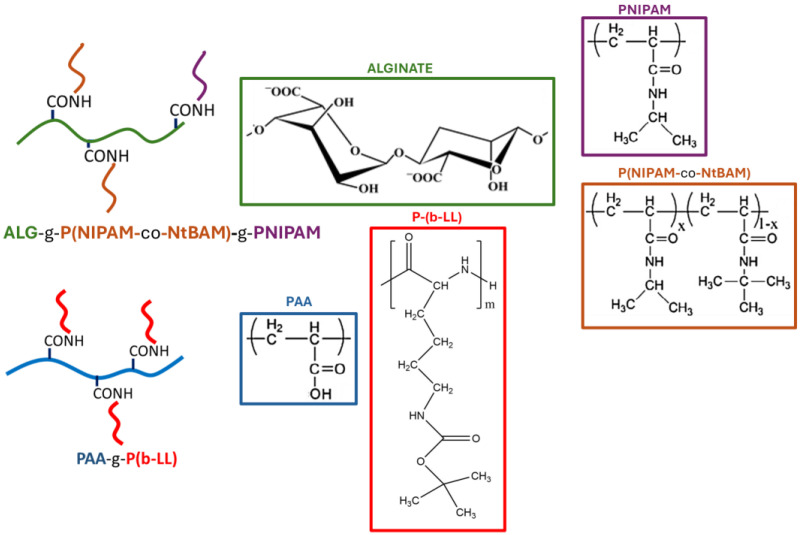
Schematic representation and chemical structures of the involved graft copolymer gelators.

In the present article, we report the study of a two-gelator composite system (see Scheme 1) constituted mainly of the alginate-based thermoresponsive heterografted copolymer, ALG-g-P(NIPAM_86_-co-NtBAM_14_)-g-PNIPAM [35], blended with a peptide-based, thermostable graft copolymer, Poly(Acrylic Acid)-g-Poly(boc-L-Lysine) (PAA-g-P(b-LL)) [38]. The former gelator (abbreviated in the following as ALG-g-HG) exhibits sol-gel transition at about 30 °C, which is controlled by the overall NtBAM content of the different side chains [35], and the latter forms thermostable shear-induced injectable hydrogel through the hydrophobic intermolecular association of the Poly(boc-L-Lysine) hydrophobic side stickers [38]. Thanks to the pH-dependent gel properties of both gelators, the investigation was performed strictly at the physiological pH of 7.4. The main outcome of the present study is that small substitutions of the main component of the blend, ALG-g-HG (5 wt%), by PAA-g-P(b-LL) (up to 1 wt%) impose remarkable effects on the thermoresponsive profile and mechanical properties of the hydrogel. Provided that both gelators exhibit polyelectrolyte characteristics, since they bear carboxylic pendant groups, ionic strength is another factor that influences the composite hydrogel properties.

## 2. Materials and Methods

### 2.1. Materials

The graft copolymers ALG-g-HG and PAA-g-P(b-LL) involved herein were synthesized by grafting onto a method using carbodiimide chemistry. Details of their synthesis and characterization are reported in references [35,38], respectively. Their molecular characteristics are reported in Table 1.

### 2.2. Hydrogel Sample Preparation

Aqueous mixtures of ALG-g-HG and PAA-g-P(b-LL) were prepared at different concentrations and salt concentrations, with pH maintained at 7.4 (see Table S1 in Supplementary Materials). The samples were homogenized using a Vortex mixer machine and a Sigma 2K15 centrifugation machine (2000–5000 rpm and 25 °C) and left to fully dissolve on a lab shaker for two days. The solute for all the samples was a phosphate buffer solution (PB) Na_2_HPO_4_/KH_2_PO_4_ (PB) 1 mM and pH = 7.4, and the pH value was controlled by adding NaOH solution (1 M). For examination of the salt effect, the mixtures with specific ionic strength were formed by adjusting the NaCl concentration in the solute (PB).

### 2.3. Rheology

The rheological studies of the hydrogel mixtures were conducted using a stress-controlled AR-2000ex (TA Instruments, New Castle, DE, USA) rheometer, with a cone and plate geometry (diameter 20 mm, angle 3°, truncation 111 µm), equipped with a Peltier plate system that allows the precise control of temperature (±0.1 °C) and a solvent trap that prevents changes in concentration due to water evaporation. The temperature sweep measurements were contacted in the linear viscoelastic regime (γ = 0.1%), frequency 1 Hz, and the increasing rate was set to 1 °C/min.

## 3. Results and Discussion

### 3.1. Composition Effect

As reported previously, the PAA-g-P(b-LL) self-assembles spontaneously in aqueous media, triggered by the hydrophobic association of the pendant P(b-LL) (stickers), forming densely packed swollen microgels, due to the highly hydrophobic nature of the stickers. Macroscopically, it forms a free-standing hydrogel with high elastic modulus, on the order of 10^3^ Pa, at a polymer concentration of 1 wt%. Hence, the motivation for this study was to use this associative graft copolymer as a reinforcing agent of the thermoresponsive ALG-g-HG hydrogel through blending. Given that the polymer concentration of the aqueous formulation influences the gel properties to a remarkable degree, the overall concentration of the components in the formulation was kept constant at 5 wt%. Thus, part of the ALG-g-HG was substituted by relatively small amounts of PAA-g-P(b-LL) (up to 1 wt%), keeping the formulation rich to ALG-g-HG, to maintain the thermoresponsive character of the hydrogel. Moreover, the pH was kept constant at 7.4.

**Figure 1:**
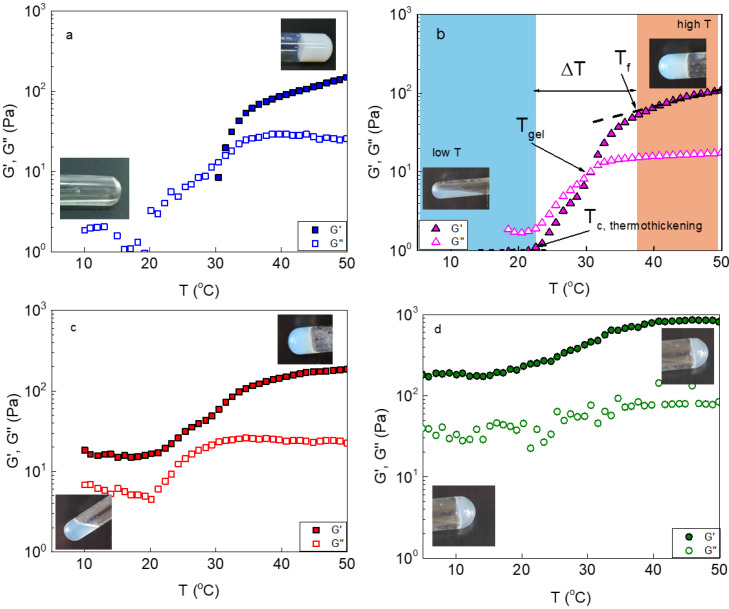
Storage (G′) (closed) and loss (G″) (open) modulus (γ = 0.1%, 1 Hz) as a function of the temperature for the ALG-g-HG/PAA-P(b-LL) systems designated in wt% of the components, (**a**) 5/0 wt%, (**b**) 4.5/0.5 wt%, (**c**) 4.25/0.75 wt% and (**d**) 4/1 wt%. In the insets, the photos show the formulations at T = 15 °C (left down) and T = 50 °C (right up).

Oscillatory rheological experiments were conducted to explore firstly the effect of the composition of the blend on the response of the temperature variations. Thus, temperature sweep measurements were conducted up to 50 °C at a rate of 1 °C/min, in the linear viscoelastic region (at γ = 0.1%) established by strain sweep tests. Figure 1 demonstrates the storage (G′) and loss (G″) modules as a function of temperature for the four formulations examined. For the sake of comparison, Figure 1a presents the thermoresponsive profile of the pure ALG-g-HG, showing a sharp increase in G′ which dominates G″ above 30 °C, revealing the formation of a hydrogel. Macroscopically (photos in Figure 1a inset), the transparent liquid at low temperatures turned to opaque free-standing gel approaching T = 50 °C.

The underpinning network is formed by the intermolecular hydrophobic association of the PNIPAM-based side chains of the graft copolymers. Upon substituting part of ALG-g-HG with PAA-g-P(b-LL), remarkable changes in the thermoresponsive profile of the hydrogel were observed. The sol-gel property of the formulations vanishes when the percentage of PAA-g-P(b-LL) exceeds 0.5 wt%, given that G′ predominates G″ in the entire temperature range explored (Figure 1c,d). Instead, a soft-gel-to-strong-gel thermo-induced transition was observed, implying that PAA-P(b-LL) can form a network of swollen microgels (G′ > G″) at temperatures lower than the critical association temperature of the LCST-type side chains of the ALG-g-HG-rich component of the blend.

Their macroscopic behavior is shown by photos in the insets of Figure 1. Moreover, the hydrogels became opaque in the entire range investigated (see Figure S2) due to the presence of PAA-g-P(b-LL) microgels formed by the P(b-LL) hydrophobic association. Notably, frequency sweep tests at 10 °C showed G′ to be nearly frequency independent at low frequencies, suggesting gel-like behaviour (Figure S3). Upon heating and above a critical temperature, the network strengthens as G′ augments remarkably, due to the activation of the LCST-type side chain hydrophobic association.

**Figure 2:**
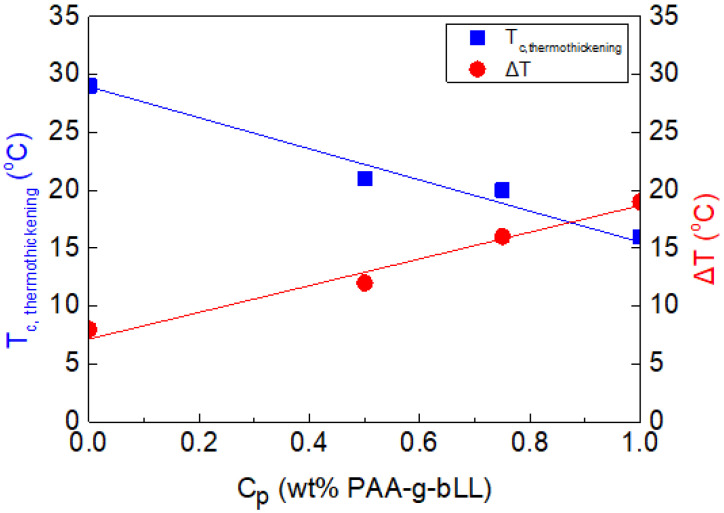
T_c,thermothickening_ and transition zone ΔΤ as a function of PAA-g-P(b-LL) component polymer concentration. The lines guide the eye.

To quantify these changes, we have defined the following critical temperatures (see Figure 1b): T_gel_ at G′ = G″; T_c,thermothickening_ above which G′ augments sharply, denoting the onset of hydrophobic association of the PNIPAM-based side chains; and T_f_ when G′ becomes independent of T or further increases smoothly. Moreover, the transition zone is the temperature interval ΔΤ = T_f_ − T_c,thermothickening_ (Table 2). In Figure 2, T_c,thermothickening_ and ΔΤ have been plotted as a function of PAA-g-P(b-LL) percentage. Two interesting effects can be observed. T_c,thermothickening_ decreased and ΔΤ increased upon blend enrichment with the PAA-g-P(b-LL) component.

In particular, T_c,thermothickening_ decreased by about 15 °C when the PAA-g-P(b-LL) component reached 1 wt%. As is known, T_c,thermothickening_ is correlated with the LCST of the side chains of the ALG-HG-rich component of the blend above which the hydrophobic association is activated [35]. As has been reported, the presence of hydrophobic moieties along the PNIPAM side chains resulted in the LCST decreasing [32,35]. Thus, we suggest that the T_c,thermothickening_ decline could be attributed to an increasingly hydrophobic environment of the side chains. This likely implies that the thermo-induced PNIPAM-based side chain association is influenced by the presence of the hydrophobic P(b-PLL) side chains of PAA-g-P(b-LL). Hence, it is reasonable to assume that some hydrophobic crosslinking domains contain mixtures of both types of stickers. Moreover, as the T_c,thermothickening_ decreased, simultaneously the transition zone ΔΤ was broadened, which suggests that the crosslinking domains formed by blending of the P(NIPAM_86_-co-NtBAM_14_), PNIPAM, and P(b-LL) side chains differ in their composition and that the domains richer in the hydrophobic P(b-LL) will broaden the transition zone toward lower temperatures.

**Figure 3:**
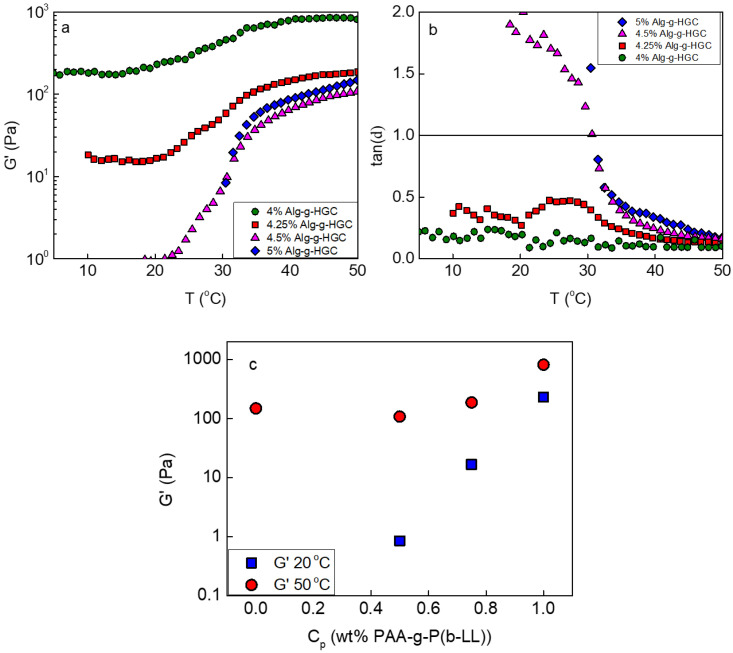
(**a**) Storage modulus (G′), and (**b**) tan(δ) as a function of temperature for the ALG-g-HG/PAA-g-P(b-LL) system at various compositions: 5/0 wt% (blue symbols); 4.5/0.5 wt% (pink symbols); 4.25/0.75 wt% (red symbols); 4/1 wt% PAA-g-P(b-LL) (green symbols). (**c**) Storage modulus (G′) at 20 and 50 °C, obtained from Figure 3a as a function for the PAA-g-P(b-LL) component polymer concentration.

To investigate the effect of incorporating of the PAA-g-P(b-LL) component to the hydrogel strength, we demonstrate in Figure 3 the temperature dependence of the elastic part of the complex modulus, G′ (Figure 3a), together with the loss tangent (G″/G′) (Figure 3b). For the sake of discussion, we can distinguish three temperature regimes: the low temperature zone (low T), below T_c,thermothickening_; the transition zone (ΔT); and the high temperature zone (high T), above T_f_ (see Figure 1b). Given that the elastic component of the complex modulus, G′, is correlated to the number density of the elastically active chains through the rubber elasticity theory, [39] changes on G′ reflect variations in the crosslinking density of the network. Figure 3a indicates that G′ varied remarkably as the percentage of the PAA-g-P(b-LL) component increased. More precisely, G′ increased about two orders of magnitude when PAA-g-P(b-LL) enhanced from 0.5 to 1 wt%, in the low T zone. Provided that the potential sticky side chains have not been activated below T_c,thermothickening_, the network formed is due to the hydrophobic association of the P(b-LL) side chains of PAA-g-P(b-LL) [38]. It is suggested that a semi-interpenetrated network was formed, comprising a PAA-g-P(b-LL) network of swollen microgels, incorporating unassociated ALG-g-HG chains. In addition, tanδ approaches about 0.2 at 1 wt% PAA-g-P(b-LL), in the low T zone, implying the formation of a hydrogel with increasing strength. In the transition zone, G′ augmented abruptly due to further association of the thermoresponsive stickers of the ALG-g-HG in all cases since the temperature surpass T_c,thermothickening_. However, in the formulations richer to PAA-g-P(b-LL), the degree of G′ increase was weakened. Less pronounced effects were observed in the high T zone, where G′ increased with the PAA-g-P(b-LL) percentage above 0.5 wt%.

In Figure 3c, G′ measured at 20 °C (low T zone) and at 50 °C (high T zone) are depicted as a function of PAA-g-P(b-LL) percentage to better demonstrate the effects discussed above. Comparing G′ values between 20 and 50 °C, we also observe that the extent of the thermothickening effect, exemplified by the ratio R_Tthick_ = G′(50)/G′(20) (Table 2), differs remarkably in the various formulations. Indeed, R_Tthick_ decreased significantly with the PAA-g-P(b-LL) percentage in the polymer blend. Particularly, this ratio decreased from 108 to 4.4 when the PAA-g-P(b-LL) percentage doubled, from 0.5 to 1 wt%, implying significant weakening of the thermothickening effect. This suggests that a smaller number of thermoresponsive side chains are still available for hydrophobic association at higher temperatures. There are two reasons for this effect. Firstly, the amount of ALG-g-HG diminishes in the same proportion as that of PAA-g-P(b-LL) increase in the blend, and secondly, part of the thermoresponsive side chains has been incorporated within the P(b-LL) hydrophobic domains of the PAA-g-P(b-LL) network, which does not contribute to the crosslinking density enhancement. Therefore, the substitution of part of ALG-g-HG by PAA-g-P(b-LL) increases remarkably the overall elasticity of the complex hydrogel but at the same time weakens the thermothickening effect.

**Figure 4:**
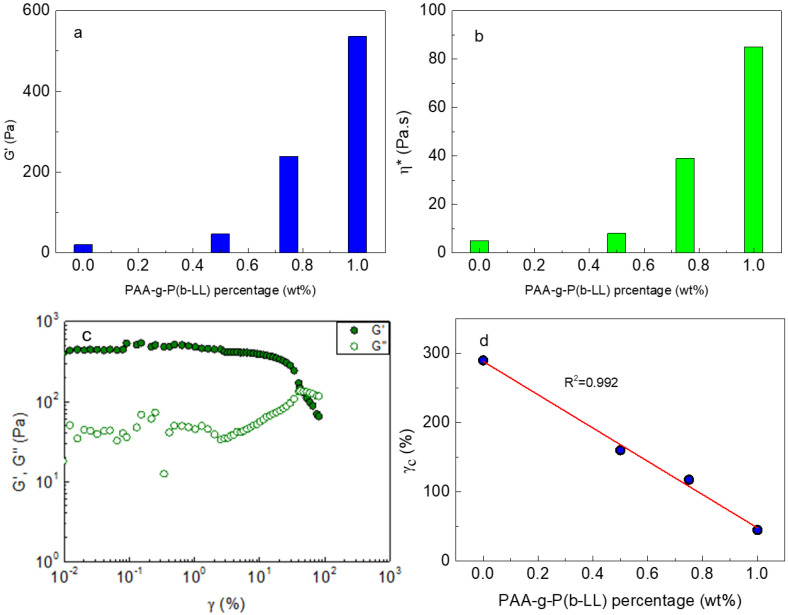
G′ (**a**), η* (**b**) and critical strain, γ_c_ (**d**) obtained from strain sweep data at 37 °C in the linear viscoelastic regime versus PAA-g-P(b-LL) percentage. (**c**) Strain sweep data of the ALG-g-HG/ PAA-g-P(b-LL) 4/1 wt% formulation.

For potential bioapplications, interest is focusing on the hydrogel properties at the physiological temperature, 37 °C. In Figure 4a,b, the storage modulus, G′, and the complex viscosity, η*, obtained from strain sweep data at 37 °C in the linear viscoelastic regime (e.g., Figure 4c), are plotted versus the PAA-g-P(b-LL) percentage. Obviously, G′ and η* increased nearly exponentially with the addition of PAA-g-P(b-LL) acting as a reinforcing agent. The strain sweep data can also give the critical strain γ_c_ at the crossover point (G′ = G″), beyond which the loss modulus predominates, revealing a strain-induced gel-sol transition. Figure 4d demonstrates a linear decrease in γ_c_ with the PAA-g-P(b-LL) percentage. Particularly, γ_c_ declined from 290%, in pure ALG-g-HG, down to 44% on the 1 wt% substitution with PAA-g-P(b-LL), revealing that the hydrogel resistance to flow weakens substantially. Provided that the pure PAA-g-P(b-LL) exhibits very low γ_c_ < 10% (which is polymer concentration dependent, see [38]), these data unveil the predominant strong influence of the PAA-g-P(b-LL) agent on the structure of the composite hydrogel. Finally, frequency sweep experiments (Figure S1) in the linear viscoelastic regime at various temperatures show G′ predominating over G″ in all frequencies, suggesting long terminal relaxation times even at low temperatures for the formulations with PAA-g-P(b-LL) percentage > 0.5 wt%.

**Figure 5:**
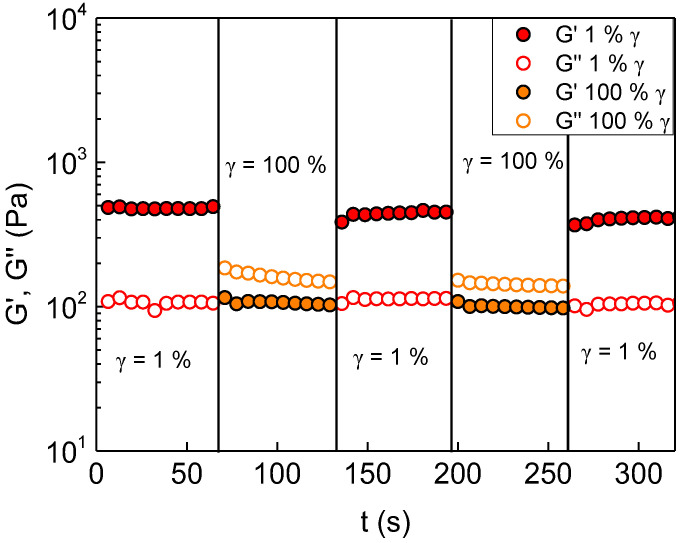
Time dependence of G′ (solid symbols) and G″ (open symbols) (1 Hz), subjected to consecutive variations in strain amplitude (as indicated), for the ALG-g-HG/PAA-g-P(b-LL) 4/1 wt% formulation at T = 37 °C and pH 7.4.

Another interesting issue relevant to the hydrogel applications is its ability to self-heal after damage imposed by a drastic stress. To explore this capability, the hydrogel was subjected to stepwise (60 sec per step) consecutive variations of strain amplitude at values of 1% (linear regime) and 100% (nonlinear regime) at 37 °C as dictated by the strain sweep data of Figure 4c. In Figure 5, time sweep oscillatory rheological data, in terms of G′, G″, obtained at 37 °C, are demonstrated, revealing that the hydrogel recovers its gel properties (G′ > G″) instantly, after imposing high stress, provoking the formulation flow (G″ > G′). Similar results were obtained with formulations of different composition (see Figure S4 in Supplementary Materials).

**Figure 6:**
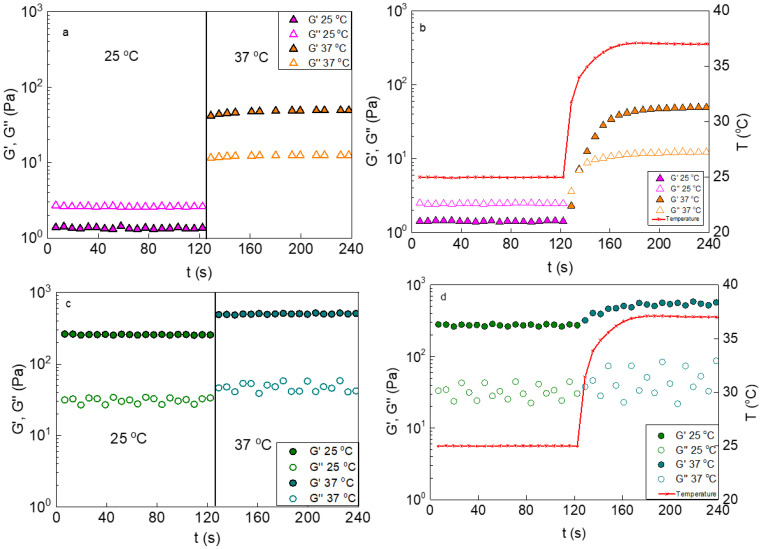
Time dependence of G′ (solid symbols) and G″ (open symbols) (γ = 0.1%, 1 Hz) for (**a**,**c**) stepwise and (**b**,**d**) fast gradual temperature variation from 25 °C to 37 °C for the 4.5 wt% ALG-g-HG/0.5 wt% PAA-g-P(b-LL) (**a**,**b**) and 4 wt% ALG-g-HG/1 wt% PAA-g-P(b-LL) (**c**,**d**) formulations.

We also examined the response to a temperature jump from room temperature to physiological temperature. Oscillatory time sweep experiments were conducted in the linear regime and 1 Hz frequency by applying a sudden change in temperature from 25 to 37 °C. Two types of tests were performed: stepwise, where the measurements at 37 °C were obtained after equilibration of temperature, and fast gradual temperature variation up to 37 °C, capturing the evolution of G′, G″ with the fast variation in temperature. In Figure 6a,b, the data concern the 4.5 wt% ALG-g-HG/0.5 wt% PAA-g-P(b-LL) formulation, which exhibits sol-gel transition upon heating (Figure 1b). As observed in Figure 6a, the sol behavior at 25 °C (G″ > G′) is instantly transformed to gel (G′ > G″) at 37 °C. In the second experiment, we can observe again the immediate response to temperature, capturing also the gel-sol transition (G′ = G″) during the fast temperature transition. On the other hand, the data of Figure 6c and d concerning the 4 wt% ALG-g-HG/1 wt% PAA-g-P(b-LL) formulation exhibits gel strengthening from 25 to 37 °C as expected from Figure 1d. In this case the changes of the moduli were instantaneous too, responding to the temperature transition. Therefore, all the results of Figure 6 show excellent thermoresposiveness of the complex hydrogel irrespective of the constituent composition.

**Figure 7:**
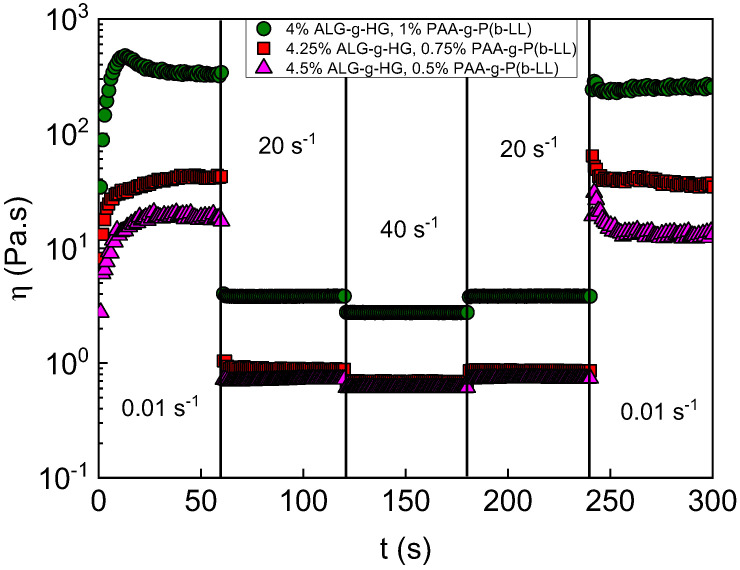
Time dependence of apparent shear viscosity after consecutive stepwise variations of shear rates of the composite hydrogels at 18 °C.

As we discussed previously, the composite hydrogel loses its sol-gel behaviour when the PAA-g-P(b-LL) reinforcing agent exceeds 0.5 wt%, meaning that the formulation exhibits gel properties even at room temperature, which raises queries about injectability. To address this issue, shear flow experiments were designed to investigate the shear responsiveness of the hydrogel. In particular, the hydrogel was subjected to stepwise sudden changes in the applied shear rate, monitoring the time-dependent shear viscosity, which is a factor relevant to injection. Figure 7 presents time sweep data under various shear rates, as indicated, for all the composite hydrogels. As seen, the shear viscosity dropped by about two orders of magnitude by increasing the shear rate from 0.01 s^−1^ to 20 s^−1^, and further decreased to 40 s^−1^. When the shear rate variation was modest, e.g., from 20 to 40 s^−1^, the response to shear viscosity was instantaneous. Importantly, upon decreasing the shear rate back to 0.01 s^−1^, the hydrogel viscosity was recovered, implying that the network structure was recovered to its initial state. Note that steady state conditions were attained within the 60 sec period of each step in all cases, confirming the prompt network structure’s recoverability. Provided that the shear rate applied through the needle of a syringe is very high (several thousand s^−1^), the shear-thinning response of the hydrogel to abrupt changes of shear, demonstrated in Figure 7, implies injectability and printability.

### 3.2. Salt Effect

**Figure 8:**
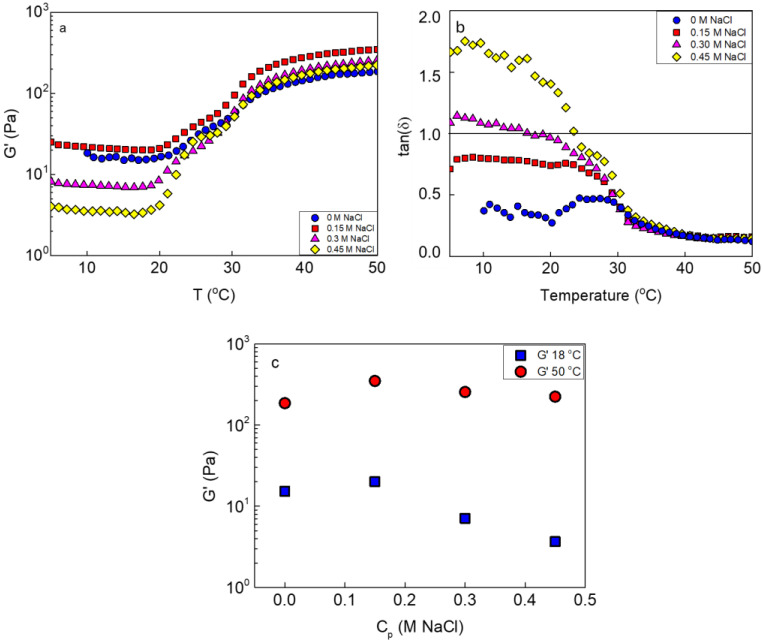
Storage modulus, G′ (**a**) and tan(δ) (**b**) (γ = 0.1%, 1 Hz) as a function of temperature for the 4.25 wt% ALG-g-HG/0.75 wt% PAA-g-P(b-LL) system at various salt NaCl concentrations: 0 M NaCl (blue symbols); 0.15M NaCl (red symbols); 0.3 M NaCl (pink symbols); 0.45 M NaCl (yellow symbols) (**c**) Salt concentration dependence of storage modulus (G′) at 18 and 50 °C, as obtained from Figure (**a**).

Rheological experiments were conducted to explore the effect of ionic strength on the hydrogel thermoresponsive properties of the 4.25 wt% ALG-g-HG/0.75 wt% PAA-g-P(b-LL) formulation, by adding NaCl in various concentrations, keeping pH at 7.4. Figure 8a and Figure 8b illustrates the storage modulus, G′, and the loss tangent (G″/G′), respectively, in the linear viscoelastic regime as a function of temperature, obtained from temperature sweep measurements by applying a heating rate of 1 °C/min (see Figure S5 in Supplementary Materials). As observed, the increase in ionic strength influences the thermoresponsive profile of the hydrogel. Specifically, it affects the structure of the network mainly in the low-temperature regime where G′ decreased about four times to 0.45 M NaCl, after an initial increase to 0.15 M NaCl (Figure 8c). More importantly, the soft-gel-to-strong-gel transition altered to sol-gel behavior upon increasing the salt concentration above 0.15 M NaCl, as can be observed from the increase of tanδ, as surpassing the value of 1 at the low T region (Figure 8b). At the high T zone, G′ exhibited similar behavior, but the magnitude of G′ remained higher than that in the free salt formulation. The overall result of the increasing ionic strength is the strengthening of the thermothickening effect, since R_Tthick_ increased from 11.5 up to 74.3 when the salt concentration reached 0.45M (Table 3), which is mainly due to the transformation of the gel to sol in the low T region.

Provided that both graft copolymers of the blend comprise backbone chains which are negatively charged polyelectrolytes, the presence of small electrolytes affects the short- and long-range electrostatic interactions. As we have discussed previously, in the low T regime, the gel properties are due to the PAA-g-P(b-LL) interpolymer association. In this state, the PAA chains adopt a stretched conformation thanks to the intramolecular repulsive interactions along the chains. The presence of a low amount of salt (0.15 M NaCl) decreases the interpolymer repulsions, allowing better hydrophobic association of the P(b-LL) stickers, as deduced from the initial increase in the elastic modulus G′. At elevated ionic strength (0.45 M NaCl), the PAA chains shrink due to the polyelectrolyte screening, which imposes a diminishing of the network connectivity, allowing the formulation to flow (sol state).

The increase in the ionic strength imposes changes on the position of the critical temperatures, namely T_gel_, T_c,thermothickening_, and transition zone ΔΤ (Table 3). T_gel_ increased from 19 °C at 0.3 N NaCl to 23 °C at 0.45 N NaCl, which should be attributed to the more pronounced decrease in G′, thus crossing G″ at a higher temperature. Nevertheless, this is clearly lower than those observed in the other formulations (see Table 2). Another notable effect is the decrease in T_c,thermothickening_ with increasing ionic strength. In these last cases, the effects could be accounted for by the influence of ionic strength on the LCST of the thermoresponsive side chains of the ALG-g-HG, according to the well-known salting-out effect [13]. The decrease in T_c,thermothickening_ also imposes ΔΤ broadening, which reveals a two-step thermothickening effect in the formulation with the highest salt concentration (see Figure S6). Note that the ALG-g-HG bears two types of side chains PNIPAM and P(NIPAM_86_-co-NtBAM_14_) with different LCSTs at 32 and 22 °C, respectively (Table 1) [32]. We assume that the effect of ionic strength is more pronounced for the P(NIPAM_86_-co-NtBAM_14_) side chains, incorporating the hydrophobic moieties NtBAM, which associate first forming hydrophobic crosslinking domains at lower temperatures, well separated from the PNIPAM, associated at higher temperatures above 28 °C. Notably, in both cases, the critical temperatures shifted to lower temperature with respect to those in salt-free conditions [32] due to the salting-out effect.

**Figure 9:**
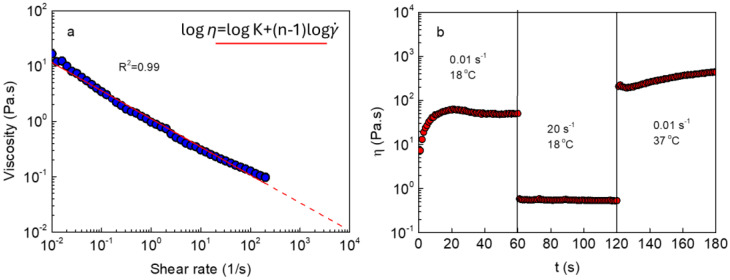
(**a**) Apparent shear viscosity versus shear rate and (**b**) time dependence of apparent shear viscosity subjected stepwise to simultaneous variations in shear rate and temperature for the 4.25 wt% ALG-g-HG/0.75 wt% PAA-g-P(b-LL) formulation in the presence of 0.15 M NaCl. The red line (extrapolated by dotted line) in (**a**) is the linear fitting according to the double log transformation of Equation (2).

The 4.25 wt%, ALG-g-HG/0.75 wt% PAA-g-P(b-LL) formulation exhibited self-healing properties at the physiological conditions of pH 7.4, T = 37 °C, and 0.15 M ionic strength (see Figure S7 in Supplementary Materials), behaving similarly to the formulation depicted in Figure 5. To explore its injection capability, flow shear experiments were conducted. In Figure 9a the shear viscosity is plotted versus shear rate, showing shear-thinning behaviour, which is one of the prerequisites for formulation injectability. The instantaneous response to shear and temperature is also necessary for injectability. This was explored by designing the following experiment, approaching an injection scheme. The hydrogel apparent shear viscosity was monitored over time in three consecutive steps under specific conditions as described in Figure 9b. In the first step, $\dot{\gamma }$ was set to 0.01 s^−1^ and T = 18 °C (gel at rest), following by a sudden change in shear rate to 20 s^−1^, keeping the same T (injection, applying high shear). Finally, in the third step, $\dot{\gamma }$ was set to 0.01 s^−1^ and T changed to 37 °C (body temperature, approaching gel at rest). As observed, the hydrogel responded instantaneously in sudden variations to shear rate and temperature-provided injectability.

Moreover, the shear rate $\dot{\gamma }$ applied during injection can be calculated using the Equation (1) [40].

(1) $$\dot{\gamma }=\frac{4Q_{v}}{\pi R_{n}^{3}}$$

where *Q_v_* and *R_n_* are the applied flow rate and the internal radius of the needle, respectively. For instance, using a 27G syringe (*R_n_* = 0.105 mm) and *Q_v_* = 1 mL/min $\dot{\gamma }$ calculated 18.35 × 10^3^ s^−1^. Applying the well-known Ostwald–de Waele Equation (2) to the data in Figure 9a, we could evaluate the value of shear viscosity during injection under this shear rate, which is η = 0.007 Pa·s. The applied injection force F can be calculated as F = Kη, where the constant K depends on the needle dimensions and *Q_v_* (see Supplementary Materials). For a 27G syringe, F calculated 0.56 N, which is well below the limit (12 N) for a comfortable injection [41]. Thus, the explored formulation is suitable for painless injectability into living bodies.

(2) $$\eta =K\;{\dot{\gamma }}^{n-1}$$

It is interesting to mention a previous study, published by our group recently [42]. That study dealt with the same thermoresponsive heterografted copolymer ALG-g-P(NIPAM_86_-co-NtBAM_14_)-g-PNIPAM, incorporating micellar nanoparticles, formed by association of poly(2-vinyl pyridine)-b-poly(ethylene oxide) (P2VP-*b*-PEO) block copolymers. The composition of the aqueous formulation was 4 wt% ALG-HG/1 wt% P2VP-*b*-PEO, and the investigation was conducted by varying the pH from 5.4 to 3.5. Surprisingly, the effect of lowering pH on the thermoresponsiveness and the rheological properties of the hydrogel was found similar to the results of the present study, i.e., transformation of sol gel to soft gel–strong gel transition, followed by a shifting of the critical temperatures to lower values, although the mechanism of network structure alterations is different. Interestingly, the present reinforced thermoresponsive injectable hydrogel was conducted at the physiological pH, making it advantageous for potential biomedical applications.

## 4. Conclusions

In the present article, we report the study of a two-gelator composite system, consisting of the alginate-based thermoresponsive heterografted copolymer, ALG-g-HG, blended with a peptide-based, thermostable graft copolymer, PAA-g-P(b-LL). The motivation of this study was to use this PAA-g-P(b-LL) as a reinforcing agent for the ALG-HG hydrogel through blending. Thus, part of the ALG-g-HG (5 wt%) was substituted by relatively small amounts of PAA-g-P(b-LL) (up to 1 wt%), keeping the formulation rich in ALG-g-HG, to maintain the thermoresponsive character of the hydrogel. The study was conducted at constant pH 7.4. Oscillatory rheological measurements revealed that the thermoresponsive profile of the hydrogels was influenced to a remarkable degree as the PAA-g-P(b-LL) percentage increased. Specifically, the thermo-induced sol-gel transition was transformed to soft gel–strong gel when the PAA-g-P(b-LL) percentage exceeded 0.5 wt%, due to the formation of a semi-interpenetrating network comprising a PAA-g-P(b-LL) network, incorporating ALG-g-HG unassociated chains at low temperatures. Moreover, the thermo-induced association of the PNIPAM-based side chains continued to be activated at higher temperatures, but the thermothickening effect was weakened. Importantly, the thermo-responsive characteristics, namely T_c,thermothickening_ and ΔT (transition temperature window), shifted and broadened to lower temperatures, respectively. This was attributed to the influence of the hydrophobic P(b-LL) side chains on the LCST of the PNIPAM-based side chains. Concerning the mechanical properties, the hydrogel elasticity and strength increased with the PAA-g-P(b-LL) percentage in the entire temperature range investigated in all formulations, indicating that the PAA-g-P(b-LL) indeed act as a reinforcing agent. This was more pronounced at temperatures lower than T_c,thermothickening_.

The effect of ionic strength on the hydrogel properties was investigated too, revealing notable effects on thermoresponsiveness and hydrogel strength. At elevated salt concentrations, the sol-gel transition emerged again due to the polyelectrolyte screening, imposing shrinkage of the polymer backbone of the gelators and in turn decreasing network connectivity. In addition, the presence of salt influenced the thermoresponsive characteristics. T_c,thermothickening_ decreased and ΔT broadened with ionic strength. Notably, a two-step gel-sol transition was observed in the presence of 0.45 N NaCl. These results were attributed to the salting-out effect, which lowers the LCST of the PNIPAM-based side chains.

All the formulations showed self-healing capability and instantaneous response to sudden changes in shear. Especially, the 4.25 wt% ALG-g-HG/0.75 wt% PAA-g-P(b-LL) formulation under physiological conditions (pH 7.4, T = 37 °C and 0.15M ionic strength) exhibited excellent injectability, suitable for minimally invasive operations. Provided that the formulation exhibits soft gel characteristics, it could be used to protect cell transportation during injection.

## Figures and Tables

**Table 1:** Molecular characteristics of ALG-g-HG and PAA-g-P(b-LL).

Polymer	Mw (g/mol)	*T_cp_* (°C) ^a^	*N* ^b^
ALG-g-HG	262,000	-	
P(NIPAM_86_-co-NtBAM_14_) side chains	17,000	22	3.5
PNIPAM side chains	22,700	32	1.8
PAA-g-P(b-LL)	581,780	-	
PAA backbone	450,000	-	
P(b-LL) side chains	11,980	-	11

^a^ cloud point (see Supplementary Materials), ^b^ average number of side chains per backbone.

**Table 2:** Characteristic factors of thermoresponsiveness of hydrogels with different ALG-g-HG/PAA-g-P(b-LL) composition.

Sample ALG-g-HG/PAA-g-P(b-LL) (wt%)	5.0/0 S2	4.5/0.5 S3	4.25/0.75 S4	4.0/1.0 S5
*Tgel* (°C)	30	29	-	-
Δ*Τ* (°C)	8	12	16	19
*Tc,thermothickening* (°C)	29	22	21	15
*R_Tthick_* = *G′*(50)/*G′*(20)	149	108	11.5	4.4

**Table 3:** Characteristic factors of thermoresponsiveness at various NaCl concentrations for the 4.25 wt% ALG-g-HG / 0.75 wt% PAA-g-P(b-LL) system.

Sample 4.25 wt% Alg-g-HG 0.75 wt% PAA-g-P(b-LL)	0 M NaCl S4	0.15 M NaCl S6	0.3 M NaCl S7	0.45 M NaCl S8
*Tgel* (°C)	-	-	19	23
Δ*Τ* (°C)	21	24	25	26
*Tc, thermothickening*	21	18	17	16
*R_Tthick_ =* *G′(50)/* *G′(* *20)*	11.5	16.6	36.6	74.3

## Data Availability

The raw data supporting the conclusions of this article will be made available by the authors on request.

## Supplementary Materials

The following supporting information can be downloaded at https://www.mdpi.com/article/10.3390/polym16243555/s1. Figure S1: Determination of Tcp of the side chains of ALG-g-HG; Figure S2: Photos of the 4.5 wt% ALG-g-HG/0.5 wt% PAA-g-P(b-LL) formulation obtained at various temperatures; Figure S3: Frequency sweep (γ = 0.1%,) at 10 °C; Figure S4. Time dependence of G′ (solid symbols) and G″ (open symbols) (1 Hz), subjected to consecutive variations of strain amplitude, for the ALG-g-HG/PAA-g-P(b-LL) system at various compositions: (a) 4.5 wt% ALG-g-HG/0.5 wt% PAA-g-P(b-LL); (b) 4.25 wt% ALG-g-HG/0.75 wt% PAA-g-P(b-LL), at T = 37 °C and pH 7.4; Figure S5: Temperature sweep (γ = 0.1%, 1 Hz) at various salt NaCl concentrations: (a) 0 M NaCl; (b) 0.15 M NaCl; (c) 0.3 M NaCl; (d) 0.45 M NaCl; for the 4.25 wt% ALG-g-HG/0.75 wt% PAA-g-P(b-LL) systems; Figure S6: Storage (G′) modulus versus temperature for the 4.25 wt% ALG-g-HG/0.75 wt% PAA-g-P(b-LL) system at (a) 0 M NaCl and (b) 0.45 M NaCl; Figure S7: Time sweep (1 Hz), subjected to consecutive variations of strain amplitude, for the 4.25 wt% ALG-g-HG/0.75 wt% PAA-g-P(b-LL) system at the physiological conditions pH 7.4, T = 37 °C and 0.15 M NaCl; Calculation of injection force F. Table S1: Characteristics of the samples. Reference [43] is cited in the Supplementary Materials.
