# Supplementary material for: Alginate Heterografted Copolymer Thermo-Induced Hydrogel Reinforced by PAA-g-P(boc-L-Lysine): Effects on Hydrogel Thermoresponsiveness
Source: Polymers (Basel). 2024 Dec 20;16(24):3555. doi: 10.3390/polym16243555 (PMC11679975; doi:10.3390/polym16243555)
Supplement: Supplementary file 1 [file polymers-16-03555-s001.zip › polymers-3376511-supplementary.pdf]

Supplementary Materials

Alginate heterografted copolymer thermo-induced hydrogel reinforced by PAA-g-P(boc-L-Lysine): Effects on Hydrogel Thermoresponsiveness.

Aikaterini-Ariadni Moschidi, Constantinos Tsitsilianis*

Department of Chemical Engineering, University of Patras, 26500 Patras, Greece

Table S1. Characteristics of the samples.

Sample	Concentration of PAA-g-P(b-LL) (wt%)	Concentration of ALG-g-HG (wt%)	Ionic Strength (M)
S1	1	0	-
S2	0	5	-
S3	0.50	4.50	-
S4	0.75	4.25	-
S5	1	4	-
S6	0.75	4.25	0.15
S7	0.75	4.25	0.30
S8	0.75	4.25	0.45

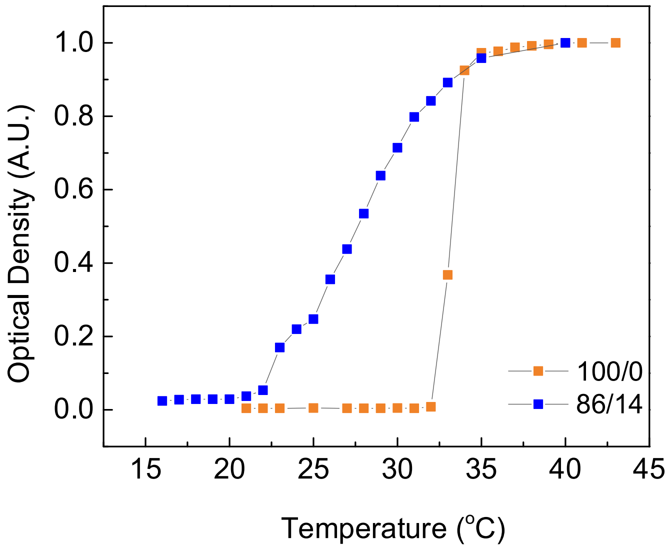

Figure S1. Temperature dependence of the normalized optical density at 500 nm of 0.5% w/v aqueous solutions of PNIPAM (orange) and P(NIPAM$_{86}$-co-NtBAM$_{14}$) (blue).

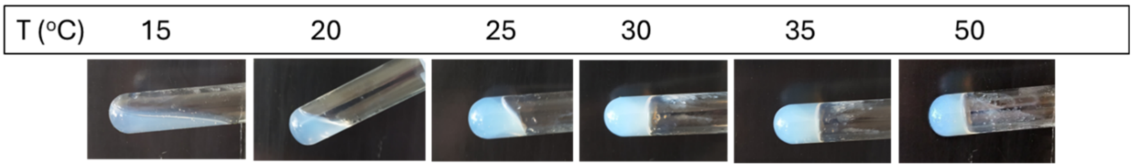

Figure S2. Photos of the 4.5 wt% ALG-g-HG/0.5 wt% PAA-g-P(b-LL) formulation obtained at various temperatures

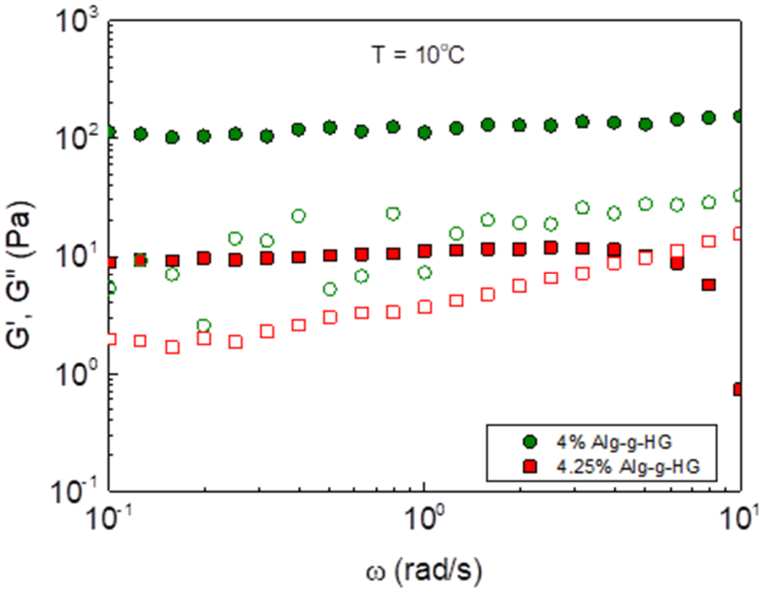

Figure S3. Storage (G') and loss (G'') moduli ($\gamma = 0.1\%$,) as a function of frequency at 10 °C, for the formulations indicated in the inset

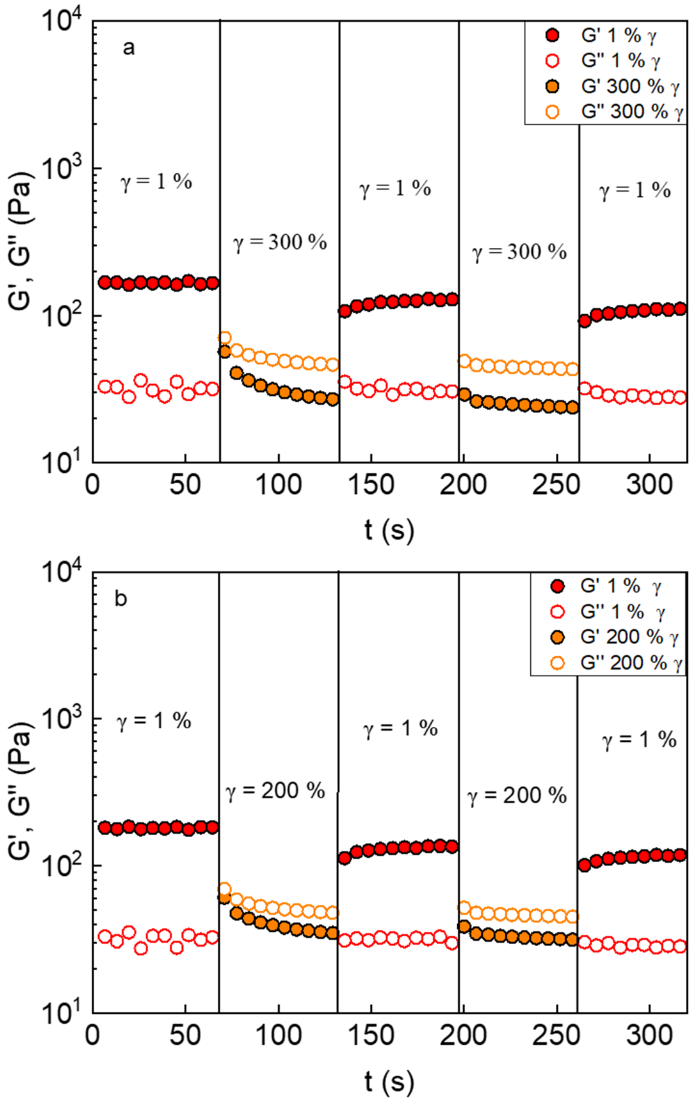

Figure S4. Time dependence of G' (solid symbols) and G" (open symbols) (1 Hz), subjected to consecutive variations of strain amplitude, for the ALG-g-HG/PAA-g-P(b-LL) system at various compositions: (a) 4.5 wt% ALG-g-HG/0.5 wt% PAA-g-P(b-LL); (b) 4.25 wt% ALG-g-HG/0.75 wt% PAA-g-P(b-LL), at T = 37 °C and pH 7.4

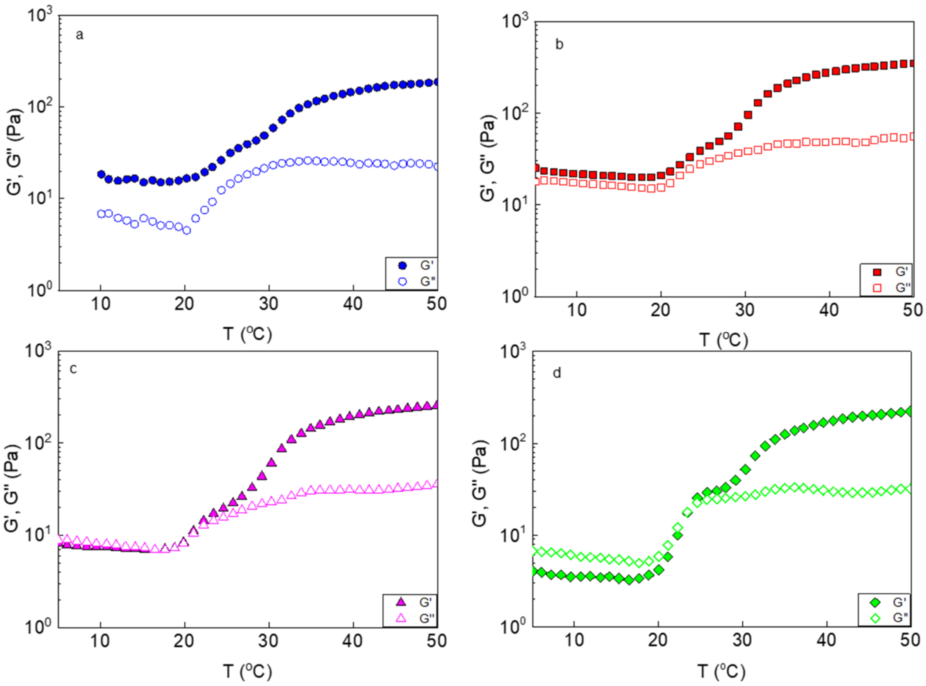

Figure S5. Storage (G’) and loss (G”) moduli (γ = 0.1%, 1 Hz) as a function of the temperature at various salt NaCl concentrations: (a) 0 M NaCl; (b) 0.15 M NaCl; (c) 0.3 M NaCl; (d) 0.45 M NaCl; for the 4.25 wt% ALG-g-HG/0.75 wt% PAA-g-P(b-LL) system at pH 7.4.

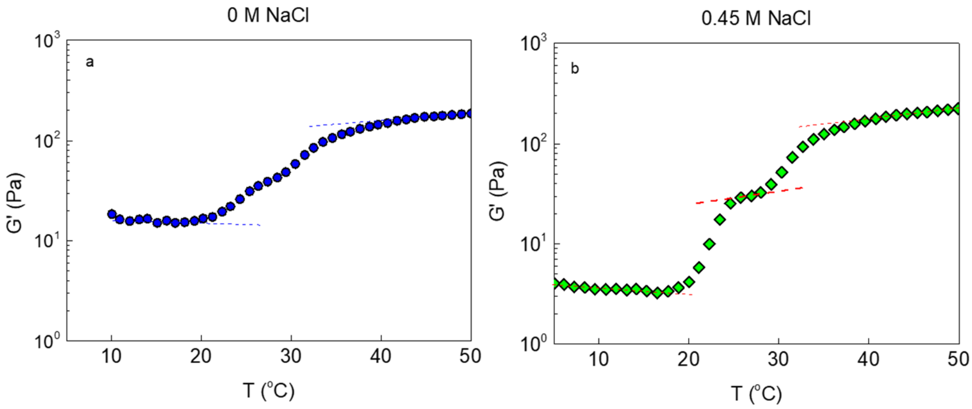

Figure S6. Storage (G’) modulus as a function of temperature for the 4.25 wt% ALG-g-HG/0.75 wt% PAA-g-P(b-LL) system at (a) 0 M NaCl and (b) 0.45 M NaCl obtained from Figure S3a and d.

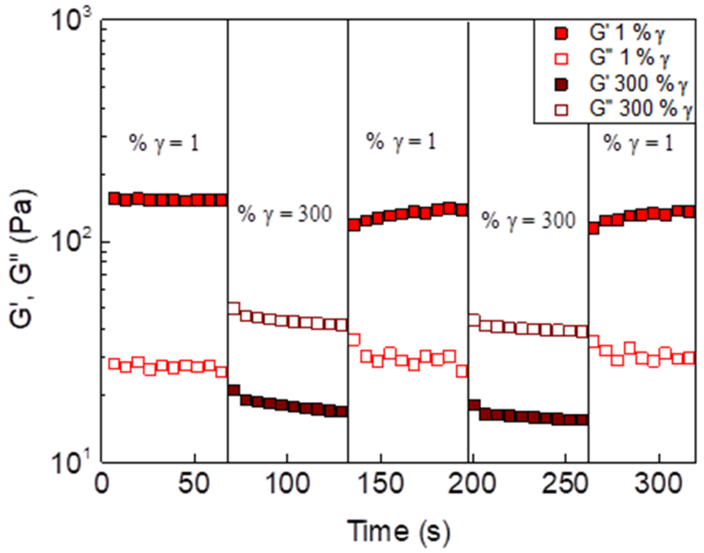

Figure S7. Time dependence of G' (solid symbols) and G'' (open symbols) (1 Hz), subjected to consecutive variations of strain amplitude, for the 4.25 wt% ALG-g-HG/0.75 wt% PAA-g-P(b-LL) system at the physiological conditions pH 7.4, T=37 °C and 0.15 M NaCl.

Injectability

The injection force F is given by the equation (1), where η is the shear viscosity and Q_v the flow rate of a liquid injected through a syringe of radius R_s, needle of radius R_n, and length L, with F_f the friction force of the piston in the syringe [1].

$$F = \frac{8\eta L Q_v R_s^2}{R_n^4} + F_f \quad (1)$$

For a 27G syringe: R_s=2.4 mm, R_n=0.105 mm, L=12.7 mm. Considering that the friction force is negligible (F_f=0) and applying Q_v=1mL/min, equation (2) can be written as F=Kη where K=80.24 m^2 s^{-1}

[1] A. Allmendinger, S. Fischer, J. Huwyler, H.-C. Mahler, E. Schwarb, I. E. Zarraga and R. Mueller, *Eur. J. Pharm. Biopharm*, **2014**, 87, 318–328.
